# *Kaempferia parviflora* Extracts Protect Neural Stem Cells from Amyloid Peptide-Mediated Inflammation in Co-Culture Model with Microglia

**DOI:** 10.3390/nu15051098

**Published:** 2023-02-22

**Authors:** Piya Temviriyanukul, Anchana Chansawhang, Jirarat Karinchai, Sataporn Phochantachinda, Shutipen Buranasinsup, Woorawee Inthachat, Pornsiri Pitchakarn, Boonrat Chantong

**Affiliations:** 1 Food and Nutrition Academic and Research Cluster, Institute of Nutrition, Mahidol University, Salaya, Phuttamonthon, Nakhon Pathom 73170, Thailand; 2 The Center for Veterinary Diagnosis, Faculty of Veterinary Science, Mahidol University, Salaya, Phutthamonthon, Nakhon Pathom 73170, Thailand; 3 Department of Biochemistry, Faculty of Medicine, Chiang Mai University, Chiang Mai 50200, Thailand; 4 Department of Clinical Sciences and Public Health, Faculty of Veterinary Science, Mahidol University, Salaya, Phutthamonthon, Nakhon Pathom 73170, Thailand; 5 Department of Pre-clinical and Applied Animal Science, Faculty of Veterinary Science, Mahidol University, Salaya, Phutthamonthon, Nakhon Pathom 73170, Thailand

**Keywords:** Alzheimer’s disease, *Kaempferia parviflora*, microglia, neuronal differentiation, neuroinflammation

## Abstract

The existence of neuroinflammation and oxidative stress surrounding amyloid beta (Aβ) plaques, a hallmark of Alzheimer’s disease (AD), has been demonstrated and may result in the activation of neuronal death and inhibition of neurogenesis. Therefore, dysregulation of neuroinflammation and oxidative stress is one possible therapeutic target for AD. *Kaempferia parviflora* Wall. ex Baker (KP), a member of the Zingiberaceae family, possesses health-promoting benefits including anti-oxidative stress and anti-inflammation in vitro and in vivo with a high level of safety; however, the role of KP in suppressing Aβ-mediated neuroinflammation and neuronal differentiation has not yet been investigated. The neuroprotective effects of KP extract against Aβ_42_ have been examined in both monoculture and co-culture systems of mouse neuroectodermal (NE-4C) stem cells and BV-2 microglia cells. Our results showed that fractions of KP extract containing 5,7-dimethoxyflavone, 5,7,4′-trimethoxyflavone, and 3,5,7,3′,4′-pentamethoxyflavone protected neural stem cells (both undifferentiated and differentiated) and microglia activation from Aβ_42_-induced neuroinflammation and oxidative stress in both monoculture and co-culture system of microglia and neuronal stem cells. Interestingly, KP extracts also prevented Aβ_42_-suppressed neurogenesis, possibly due to the contained methoxyflavone derivatives. Our data indicated the promising role of KP in treating AD through the suppression of neuroinflammation and oxidative stress induced by Aβ peptides.

## 1. Introduction

Dementia is a type of neurodegenerative disease comprising frontotemporal dementia, Lewy body dementia, vascular dementia, and Alzheimer’s disease (AD), with AD accounting for 60–70% of all dementia cases [1]. Dementia, including AD, is characterized by neuronal loss, leading to a decline in memory and cognitive ability that negatively impact individuals and their families [1]. AD etiology is multifaceted, with several variables including genetic factors, reduced neurogenesis, mitochondrial dysfunction, oxidative stress accumulation, neuroinflammation, and accumulation of hyperphosphorylated tau and amyloid beta (Aβ) peptides. These variables interact and generate a vicious cycle that accelerates neuronal damage and death, leading to neuron loss and brain atrophy [2,3]. The neuroinflammatory responses surrounding Aβ plaques in AD suggest that inflammation and sustained immune responses activated by microglia and reactive astrocytes are disease characteristics [2,4]. 

Neural stem cells can multiply and differentiate into many cell types in the nervous system such as oligodendrocytes, microglia, and neurons. Several studies showed that Aβ peptides reduce neural stem cell survival and proliferation and decrease neurogenic differentiation [5,6,7]; thus, neurogenesis dysfunction could be considered a hallmark of AD [2,8]. Currently, the US Food and Drug Administration (FDA) has approved AD medicines including donepezil, galantamine, rivastigmine, and memantine. The first three act as cholinesterase inhibitors, while memantine acts as a blocking agent for N-methyl-D-aspartate (NMDA) receptors. However, these medicines cannot treat behavioral and psychiatric symptoms in moderate and severe stages of AD [9], and suppression of Aβ-mediated neuroinflammation with improved survival of neural stem cells is an alternative AD therapy. Ginkgo biloba extract, curcumin, garcinol, gallic acid, and ginsenosides have all been reported for their anti-AD properties through the reduction of Aβ-mediated neuroinflammation and the ability to modify the process of neural differentiation [10,11,12,13]. 

*Kaempferia parviflora* Wall. ex Baker (*K. parviflora*; KP) is a perennial plant belonging to the Zingiberaceae family, like turmeric (*Curcuma longa* L.) and ginger (*Zingiber officinale* Roscoe). The KP rhizome is mostly employed as a health-promoting substance in traditional medicine to treat a broad range of illnesses including anti-osteoarthritis, anticancer, anti-psoriasis, anti-obesity, antioxidative stress, anti-AD, and anti-inflammation [14,15,16,17,18]. An ethanolic extract of KP reduced gene and protein expression of inflammatory markers including interleukin-1β (IL-1β), interleukin-6 (IL-6), and tumor necrosis factor-α (TNF-α) in lipopolysaccharide (LPS)-stimulated macrophages through inhibition of the nuclear factor-κB (NF-κB) translocation [14], while 5,7,4′-trimethoxyflavone (TMF) and 5,7-dimethoxyflavone (DMF) isolated from KP exhibited potential enzyme inhibitory activities against acetylcholinesterase (AChE) and butyrylcholinesterase (BChE), which are drug targets for AD treatment as mentioned above [19]. Moreover, the ethanolic extract of KP containing 3,5,7,3′,4′-pentamethoxflavone (PMF), 5,7,4′-trimethoxyflavone, and 5,7-dimethoxyflavone alleviated cognitive deficits and prevented a decrease in neural progenitor cell division caused by valproic acid (VPA) administration in rats [20]. VPA is an antiepileptic medication that inhibits cell proliferation and neurogenesis in the hippocampus [21] that causes moderate to severe cognitive impairments [22]. KP has also been examined for its toxicity and effectiveness. KP extract had a no-observed-adverse-effect-level (NOAEL) of >249 mg/kg BW/day according to sub-chronic testing and was also free of genotoxicity, suggesting a high degree of safety [23]. Overall, KP extract showed promise as a natural, safe ingredient to treat anti-inflammatory and anti-AD activities by inhibiting AChE, BChE, and neurogenesis decline. Therefore, this study hypothesized that KP extract may play a role in suppressing Aβ-mediated neuroinflammation and neuronal differentiation as a novel mechanism for the prevention and/or reduction of inflammation-induced AD. This is the first investigation to determine which KP fractions can counteract Aβ effects through the activity of neural stem cells, microglial cells, and differentiated neurons. Data from single cell culture and co-culture between neuronal stem cells (NE-4C) and microglial cells (BV-2) were also compared to observe the relationship between them that partially mimicked neuroglia. Results showed that KP exerted anti-neuroinflammatory properties with neuroprotective potential and promoted neuronal differentiation against Aβ. 

## 2. Materials and Methods

### 2.1. Chemicals

Cell culture medium and supplements, such as minimum essential medium (MEM), Roswell Park Memorial Institute medium 1640 (RPMI-1640), neurobasal medium, glutamine, penicillin, streptomycin, fungizone, and trypsin, were acquired from Invitrogen (Waltham, MA, USA). B-27 and fetal bovine serum (FBS) were purchased from Thermo Fisher Scientific (Waltham, MA, USA) and HyClone (Logan, UT, USA), respectively. The IL-6 ELISA kit was purchased from Abcam (Cambridge, MA, USA). The Griess reagent and CellTiter-Glo^®^ Luminescence assay kit were purchased from Promega (Madison, WI, USA). Tri-RNA Reagent was purchased from Favorgen (Kaohsiung, Taiwan). The 2× qPCRBIO SyGreen 1step Lo-ROX was obtained from PCR Biosystems (Wayne, PA, USA). 2,3-Bis-(2-Methoxy-4-Nitro-5-Sulfophenyl)-2HTetrazolium-5-Carboxanilide (XTT), phenazine methosulfate (PMS), Aβ_42_ peptides, hexafluoropropanol, poly-l-lysine (PLL), dichlorofluorescein diacetate (DCFDA), were obtained from Sigma-Aldrich (St. Louis, MO, USA). Unless otherwise noted, all additional reagents and standards were from Sigma-Aldrich.

### 2.2. Preparation of Amyloid Beta Peptides (Aβ_42_)

Aβ_42_ was prepared using previous procedures with some modifications [24,25,26]. In brief, Aβ_42_ peptides were dissolved in hexafluoropropanol. Subsequently, the solution was aliquoted, dried in a speed-vacuum centrifuge, and stored at −80 °C. The dried peptide was reconstituted in 5 mM dimethyl sulfoxide (DMSO) and diluted with phosphate-buffered saline (PBS) to reach a concentration of 100 μM. The solution was then allowed to aggregate for 72 h at 37 °C and subsequently aliquoted and kept at −80 °C until use.

### 2.3. Plant Extraction and Characterization

*Kaempferia parviflora* Wall. Ex Baker (*K. parviflora*; KP) was purchased from a traditional Thai pharmacy in Bangkok, Thailand, in 2018. For preparing the extract, dried KP was pulverized into coarse powder and soaked with 95% ethanol for 96 h [27,28]. The filtrate was evaporated and freeze-dried to obtain the crude ethanolic KP extract (KP1). The crude extracted was dissolved in 50% ETOH, then partitioned with hexane, chloroform, and ethyl acetate, and then evaporated and freeze-dried, obtaining hexane (KP2), chloroform (KP3), ethyl acetate (KP4), and residue (KP5) fractions. The extracts were kept at −80 °C until used.

Phenolic derivatives and 5,7-dimethylflavone (DMF) contents in the extract were determined by high-performance liquid chromatography (HPLC) using a C18 column (250 mm × 4.6 mm, 5 µm) (Agilent Technologies, Santa Clara, CA, USA). Gradient elution for detection was performed using two solvents, A (1% acetic acid in water) and B (100% acetonitrile). Twenty microliters of 10 mg of KP extract and its fractions were dissolved in 1 mL of diH_2_O, injected into the column with a flow rate of 0.7 mL/min, and detected at 280 nm. The peak area and retention time of the sample were determined and compared with authentic standards including catechin, caffeic acid, rutin, rosmarinic acid, quercetin, apigenin, and kaempferol.

High-performance liquid chromatography (HPLC) was performed using the above column to determine the existence of flavone derivatives, DMF (5,7-dimethoxyflavone), PMF (3,5,7,3′,4’-pentamethoxyflavone), and TMF (5,7,4’-trimethoxyflavone) contents in the KP crude extract and its fractions. The flavone derivatives were separated using a gradient system of mobile phase A (water) and mobile phase B (100% methanol) with a total run time of 60 min and flow rate of 0.6 mL/min. The gradient system was 35% A in 0–35 min, followed by 20% A in 40–55 min and 35% A in the next 60 min. Then, 10 mg/mL of the extract dissolved in 1 mL of methanol was injected into the column and detected at 254 nm. Peak areas and retention times of the samples were evaluated and compared with the standard curve of the authentic standards (PMF, DMF, and TMF).

### 2.4. Cell Cultures

#### 2.4.1. Monoculture of Neural Stem Cells (NE-4C)

Mouse neuroectodermal (NE-4C) stem cell line (CRL-2925) was obtained and cultured according to ATCC culture method guidelines (Manassas, VA, USA). Briefly, cells were maintained in MEM supplemented with 5% FBS 2 mM glutamine, 100 units/mL penicillin, and 100 µg/mL streptomycin at 37 °C with 5% CO_2_. Cells were plated on a 0.01% PLL-coated culture flask at a cell density of 10^4^ cells/cm^2^ and subcultured at a 1:10 ratio. Supplemented MEM was changed every two days.

#### 2.4.2. Monoculture of Microglia Cells (BV-2)

BV2 cells, a mouse microglial cell line, were purchased from Interlab Cell Line Collection (ATL033001, Genova, Italy). The cells were cultured and seeded according to the previous reports [29,30].

#### 2.4.3. Differentiation of NE-4C Neural Stem Cells into Neurons

The differentiation of NE-4C stem cells into neurons was performed according to the previous protocol [31]. Briefly, NE-4C cells were dissociated with 0.05% trypsin-EDTA and plated onto PLL-precoated 24-well plates at 1.5 × 10^6^ cells/well. The cells were cultured in supplemented MEM for 12 h before neuronal differentiation induction. Differentiation was induced by replacing the MEM with a neurobasal medium supplemented with 100 units/mL penicillin, 100 µg/mL streptomycin, and B-27. The supplemented neurobasal medium was replaced every 48 h. Six days after induction, aggregates with clusters of neural stem cells were mechanically dissociated, seeded onto PLL-coated 24-well plates, and grown until day twelve.

#### 2.4.4. Co-Culture System between NE-4C Stem Cells and BV-2 Microglial Cells

Co-culture of NE-4C stem cells and microglial cells was performed using 24-well insert plates (Millipore, Burlington, MA, USA). NE-4C cells were cultured in PLL-coated 24-well plates at a density of 1.5 × 10^6^ cells/well in supplemented MEM at the bottom of the well, while microglia were cultured in supplemented RPMI at a density of 1 × 10^5^ cells/well in the insert for 24 h.

Induction of neuronal differentiation in the co-culture system between NE-4C stem cells and BV-2 microglial cells was performed by replacing the media with the supplemented neurobasal medium. The BV-2 cells were loaded for co-culture with the plated NE-4C cells and shared media of the supplemented neurobasal medium. After six days, neurospheres were collected and dissociated, seeded onto PLL- coated 24-well plates without co-cultured microglial cells, and grown until day twelve.

#### 2.4.5. Co-Culture System between Differentiated Neurons Derived from NE-4C Cells and BV-2 Microglial Cells

NE-4C stem cells were differentiated into neuronal cells and cultured in supplemented neurobasal medium for 12 days to promote neuronal maturation in the bottom of the well. Consequently, the BV-2 cells in the insert were loaded as an upper chamber for co-culture with the matured neurons and the shared media of neuronal cells.

### 2.5. Cytotoxic Effect of Amyloid Beta Peptides and KP Extracts

BV-2 and NE-4C cells were seeded in a 96-well plate at 1 × 10^4^ and 2.0 × 10^4^ cells/well, respectively, and incubated overnight using the 12-day-differentiated neuron-derived NE-4C cells at 1.5 × 10^6^ cells/well grown in a 24-well plate. Based on the previous studies, the cells were treated with various concentrations of Aβ_42_ solution (0.25, 0.5, 1, 5, 10 and 20 µM) [5,26,32,33,34,35]. The cytotoxicity of KP extract (KP1–KP5) ranges from 0.5 ng/mL^−1^ mg/mL was tested in the cells for 24 h. An equivalent quantity of 0.05% DMSO was used as a vehicle control.

### 2.6. Protective Effects of KP Extracts in Both Mono and Co-Culture

The protective effects of KP extract on Aβ_42_-induced cytotoxicity were performed for both monocultures (BV-2 cells, undifferentiated NE-4C cells and differentiated neurons) and co-cultured (BV-2 cells cultured with undifferentiated NE-4C cells, and BV-2 cells cultured with differentiated NE-4C cells) as described. Aβ_42_ at 5 µM, which reduced 50% of cellular ATP level, was cotreated with KP extracts at nontoxic concentrations (0.5, 1, 2, 4, and 8 µg/mL) and incubated for 24 h. After exposure, the XTT reduction assay and ATP level were utilized to evaluate cytotoxicity.

### 2.7. Anti-Inflammatory Activities of KP Extracts on Aβ_42_-Induced Inflammation in Monoculture

The BV-2 cells were seed in a 96-well plate at 1 × 10^4^ cells/well and co-incubated with 1 µM Aβ_42_ with or without KP extract at nontoxic concentrations (0.5, 1, 2, 4 and 8 µg/mL) for 24 h. BV-2 cells were harvested to determine the intracellular reactive oxygen species (ROS) using the DCF fluorescence assay, while the culture supernatant was collected and determined for IL-6 and nitrite levels.

### 2.8. Anti-Inflammatory Effects of KP Extracts on Aβ_42_-Induced Inflammation in Co-Culture

Two co-culture models as matured neuronal cells derived from NE-4C cells and BV-2 microglial cells, and undifferentiated NE-4C neural stem cells and BV-2 microglial cells prepared as mentioned above were used to investigate whether KP extracts could suppress Aβ_42_-induced inflammation via microglial activation. Aβ_42_ (1 µM) and extracts including KP1, KP2, and KP3 (0.5, 1, 2, 4, and 8 µg/mL) were added to the culture system medium for 24 h. The culture supernatant was collected and measured for IL-6 and nitrite levels. BV-2 cells were collected to evaluate the expression of IL-6 and iNOS mRNA and determine intracellular ROS. Differentiated neuronal cells and undifferentiated NE-4C neural stem cells were collected to determine cytotoxicity and intracellular ROS.

### 2.9. Effects of KP Extracts on Neurogenesis of Aβ_42_-Induced NE-4C Cells in Monoculture

NE-4C cells were induced for differentiation in the supplemented neurobasal medium for 24 h and then exposed to Aβ_42_ (0.25 µM) with or without KP extracts at nontoxic concentrations (0.5, 1, 2, 4, and 8 µg/mL) for six days. The differentiation medium was replaced every 48 h. At day six, cells were dissociated, seeded onto 24-well culture plates and grown until day twelve. Differentiated neuronal cells were collected for expression of βIII-tubulin and MAP-2 analysis.

### 2.10. Effects of KP Extracts on Neurogenesis in Aβ_42_-Induced NE-4C Cells in Co-Culture with BV-2 Cells

The BV-2 cells were loaded for co-culture with plated NE-4C cells for one day in the supplemented neurobasal medium. Then, Aβ_42_ (0.25 µM) with or without extracts including KP1, KP2, and KP3 (0.5, 1, 2, 4, and 8 µg/mL) was added, with differentiation continued for five days. Culture media were collected for IL-6 and nitrite level determination. BV-2 cells were collected for quantitative PCR analysis of IL-6 and iNOS expression and to determine the intracellular ROS. NE-4C cells were dissociated, seeded onto 24-well culture plates, and grown until day twelve without Aβ_42_ or KP extracts. Differentiated neuronal cells were collected for βIII-tubulin and MAP-2 mRNA analysis.

### 2.11. Sodium 3′-[1-(Phenylaminocarbonyl)-3,4-tetrazolium]-bis (4-Methoxy-nitro) Benzene Sulfonic Acid Hydrate (XTT) Reduction Assay

The cytotoxicity using an XTT-based assay was performed as previously detailed [29,30]. After the exposure times, the media was discarded. Then, 0.3 mg/mL XTT and 125 mM PMS were added. After incubation at 37 °C for 2 h, the absorbance was measured at 450 nm (SpectraMax^®^ iD3, Molecular Devices, San Jose, CA, USA). Results are expressed as a percentage of cells that were untreated as a negative control.

### 2.12. Determination of ATP Levels

The total intracellular ATP level was determined using the CellTiter-Glo^®^ Luminescence assay kit. Following cell treatment, the assay reagent containing substrate was added per each well and mixed for 30 min under light protection. The luminescence at 550 nm was measured (SpectraMax^®^ iD3, Molecular Devices, San Jose, CA, USA), and the luminescence signal was expressed as a percentage of control.

### 2.13. Interleukin-6 (IL-6) Measurement

After the treatment, the culture medium was harvested and centrifuged at 2000× *g* for 10 min at 4 °C and kept at −20 °C until analysis. The culture supernatant was submitted to an IL-6 quantitative sandwich ELISA kit. The absorbance was measured at 450 nm using a microplate reader. The concentration of IL-6 in the samples was calculated in the comparison with the standard, curve (ranging from 10 to 500 pg/mL of IL-6).

### 2.14. Nitric Oxide (NO) Measurement

Griess reagent was used to NO levels. The culture medium (50 µL) was mixed with sulfanilamide solution (50 µL) for 10 min before being incubated with napthylethylenediamine dihydrochloride solution (50 µL) for 10 min. The absorbance at 520 nm was determined by a microplate reader.

### 2.15. Intercellular Reactive Oxygen Species (ROS) Measurement

The treated cells were washed with PBS, followed by preincubation with 20 µM 2,7-dichlorofluorescein diacetate (DCFDA) in a prewarmed culture medium for 30 min at 37 °C in the dark. The supernatant was removed, and the cells were washed with PBS, followed by the addition of 200 μL of cell lysis buffer (90% dimethyl sulfoxide/10% PBS). The mixtures were incubated for 5 min. The mixture (150 μL) was then transferred to a black 96-well plate, and the fluorescence was quantitated using a fluorometric plate reader (SpectraMax^®^ iD3, Molecular Devices, San Jose, CA, USA) at 480 nm/530 nm excitation/emission wavelengths [36].

### 2.16. Reverse Transcription–Quantitative Polymerase Chain Reaction (RT–qPCR)

Total RNA was extracted from the treated cells using Tri-RNA Reagent. The RNA quality and quantity were analyzed using Nanodrop ND-1000 spectrophotometry (NanoDrop Technologies, Wilmington, DE, USA). One hundred nanograms of total RNA was added and prepared in the RT–qPCR reaction mixture, qPCRBIO SyGreen 1-step Lo-ROX. Quantitative polymerase chain reaction (qPCR) was performed using qTOWER3 Real-Time PCR Systems (Analytik Jena, Langewiesen, Germany) and then analyzed using qPCRsoft 3.4 software (Analytik Jena, Langewiesen, Germany). Relative levels of target gene expression were normalized to glyceraldehyde 3-phosphate dehydrogenase (GAPDH) mRNA using the 2^−ΔΔCT^ method [37]. Each experiment was replicated at least three times, and the primer sequences are listed in the Supplementary Table S1.

### 2.17. Statistical Analysis

Data from at least three separate experiments are reported as mean ± standard deviation (SD). GraphPad Prism 9.0 (GraphPad Software, Boston, MA, USA) was used to statistically analyze the data. A one-way ANOVA followed by Tukey’s test was used to determine the statistical significance of differences between groups. A probability of 0.05 or less (*p* ≤ 0.05) was considered statistically significant.

## 3. Results

### 3.1. KP Extraction and Phytochemical Analysis

KP extraction yielded ethanolic crude extract (KP1), hexane (KP2), chloroform (KP3), ethyl acetate (KP4), and residue (KP5) fractions as 4.67%, 18.6%, 78.0%, 0.49%, and 0.67%, respectively. KP3 extracted from chloroform contained major *K. parviflora* components, suggesting the predominance of nonpolar phytochemicals. HPLC analysis detected caffeic acid and rutin in KP4, whereas only catechin was found in KP5 (Table 1 and Supplementary Figure S1). The flavone contents of KP have been well-documented and methoxyflavone derivatives were also determined, as shown in Table 1 and Supplementary Figure S2. DMF, PMF, and TMF were predominant in KP1 and KP3, with minor amounts in KP2 and KP4. TMF was the most abundant flavone derivative found in KP1 to KP4 compared to DMF and TMF. Interestingly, these three derivatives were not detectable in the KP5 fraction.

### 3.2. Cytotoxicity Determination of Aβ_42_ and K. parviflora Extracts

**Figure 1:**
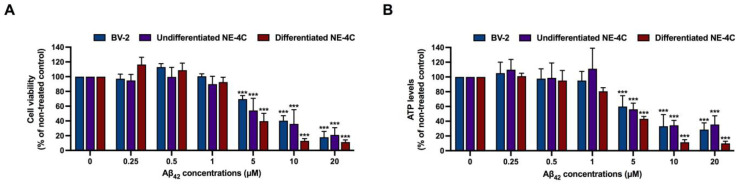
Cytotoxicity effects of amyloid beta peptides (Aβ_42_) on BV-2 cells, undifferentiated NE-4C and differentiated NE-4C. (**A**) cytotoxicity effects of Aβ_42_ measured by XTT assay and (**B**) cytotoxicity effects of Aβ_42_ measured by ATP assay. The values are mean ± SD of three independent experiments and statistical significance was analyzed against untreated control (0 µM) of each cell line using one-way ANOVA followed by Tukey’s test. ***, *p* < 0.001.

To investigate the protective effects of *K. parviflora* extract against Aβ_42_ peptides, we first determined the cytotoxic effects of Aβ_42_ peptides and the five fractions of *K. parviflora* extracts to establish the ranges of appropriate concentrations for the whole study, using XTT reduction and ATP levels as two independent cytotoxicity assays. The three cell lines, BV-2, undifferentiated NE-4C and differentiated NE-4C were treated with various ranges of Aβ_42_ and the five fractions of *K. parviflora* extracts (KP1–KP5) for 24 h. Figure 1A,B show that after 24 h of treatment, Aβ_42_ at ≤1 µM was not cytotoxic to all tested cells, while Aβ_42_ at ≥5 µM showed significant cytotoxicity toward all tested cells in our condition.

**Figure 2:**
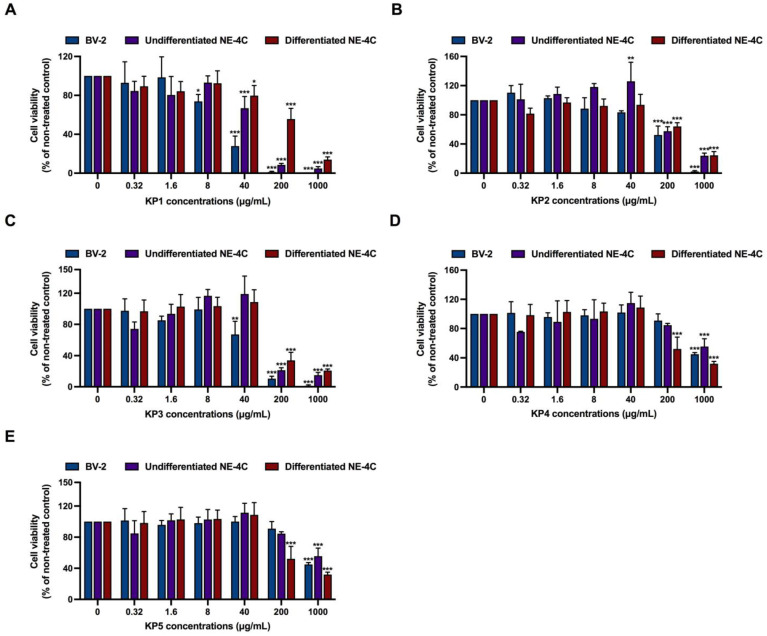
Cytotoxicity effects of *K. parviflora* extracts (KP1–KP5) on BV-2 cells, undifferentiated NE-4C and differentiated NE-4C measured by XTT assay. (**A**) KP1 fraction, (**B**) KP2 fraction, (**C**) KP3 fraction, (**D**) KP4 fraction, and (**E**) KP5 fraction. The values are mean ± SD of three independent experiments and statistical significance was analyzed against untreated control (0 µM) of each cell line by one-way ANOVA followed by Tukey’s test. *, *p* < 0.05, **, *p* < 0.01, ***, *p* < 0.001.

Results from the XTT assay also revealed that all five fractions of *K. parviflora* extracts (KP1–KP5) displayed different cytotoxic effects. In BV-2 cells, KP1 markedly showed toxicity starting from 40 µg/mL, while KP2–KP3 showed toxicity starting from 200 µg/mL and KP4–KP5 exhibited clear toxicity starting from 1000 µg/mL (Figure 2A–E). In undifferentiated NE-4C, KP1–KP3 showed toxicity starting from 200 µg/mL, and KP4–KP5 showed toxicity starting from 1000 µg/mL. Moreover, in differentiated NE-4C, significant toxicity was obtained from 200 µg/mL of KP1–KP5. Thus, 0.5 to 8 µg/mL of KP1–KP5, which were sub-cytotoxic concentrations, were selected for further investigation.

### 3.3. Protective Effects of K. parviflora Extracts on Aβ_42_-Mediated Neurotoxicity

**Figure 3:**
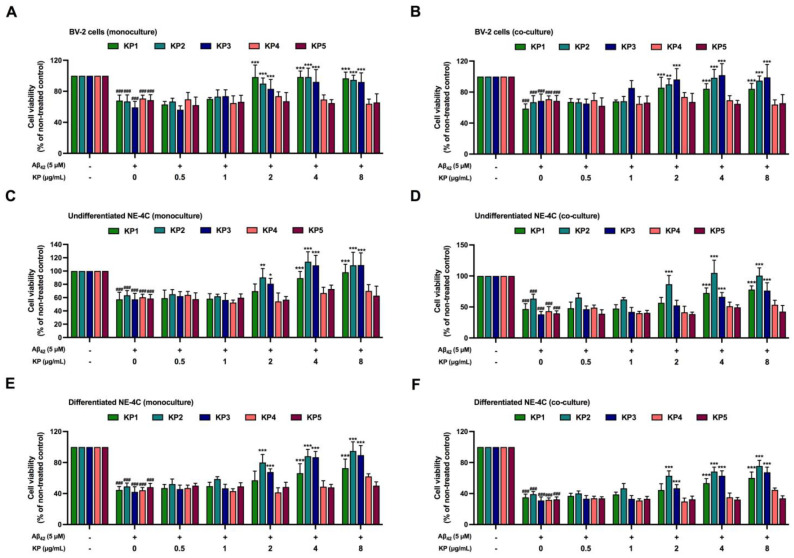
Protective effects of five *K. parviflora* extracts against Aβ_42_-induced neuronal cell death in both NE-4C monoculture and co-culture between NE-4C and BV-2 cells determined using an XTT assay. (**A**) BV-2 monoculture, (**B**) BV-2 co-culture, (**C**) undifferentiated, NE-4C monoculture, (**D**) undifferentiated NE-4C co-culture, (**E**) differentiated NE-4C monoculture, and (**F**) differentiated NE-4C co-culture. Values are mean ± SD of three independent experiments. The ^###^ indicates a significant difference between the control and cells exposed to Aβ_42_ peptides without KP extract at *p* < 0.001 using one-way ANOVA followed by Tukey’s test. The * indicates a significant difference between cells exposed to Aβ_42_ peptides without KP extract compared to cells exposed to Aβ_42_ peptides and various concentrations of KP extract (0.5–8 µg/mL) using one-way ANOVA followed by Tukey’s test. *, *p* < 0.05, **, *p* < 0.01, ***, *p* < 0.001.

The protective effects of all five *K. parviflora* extract against Aβ_42_-mediated neurotoxicity in both NE-4C monoculture and co-culture between NE-4C and BV-2 cells were evaluated. Aβ_42_ at 5 µM was selected to induce neuronal death as this mirrored the IC_50_ value, using subtoxic doses of *K. parviflora* extracts (0.5–8 µg/mL). The NE-4C monoculture or co-culture between NE-4C and BV-2 cells was treated with Aβ_42_ and *K. parviflora* extracts. After 24 h of treatment, cytotoxicity was tested using the XTT assay. Figure 3A,B show that low concentrations of all five *K. parviflora* fractions (0.5–1 µg/mL) had no protective effects against Aβ_42_, while only KP1, KP2, and KP3 at 2–8 µg/mL effectively normalized Aβ_42_-mediated neurotoxicity in both monoculture and co-culture of BV-2 cells. Interestingly, KP1 (at least 4 µg/mL), KP2 (at least 2 µg/mL), and KP3 (at least 4 µg/mL), but not KP4 and KP5 significantly suppressed Aβ_42_ toxicity in undifferentiated and differentiated NE-4C cells (both monoculture and co-culture). Therefore, KP1, KP2, and KP3 fractions exhibited protective effects against Aβ_42_-induced neuronal death in both monoculture and co-culture models.

### 3.4. Suppression of Aβ_42_-Induced Inflammation and Oxidative Stress by K. parviflora Extracts in BV-2 Cell Monoculture

**Figure 4:**
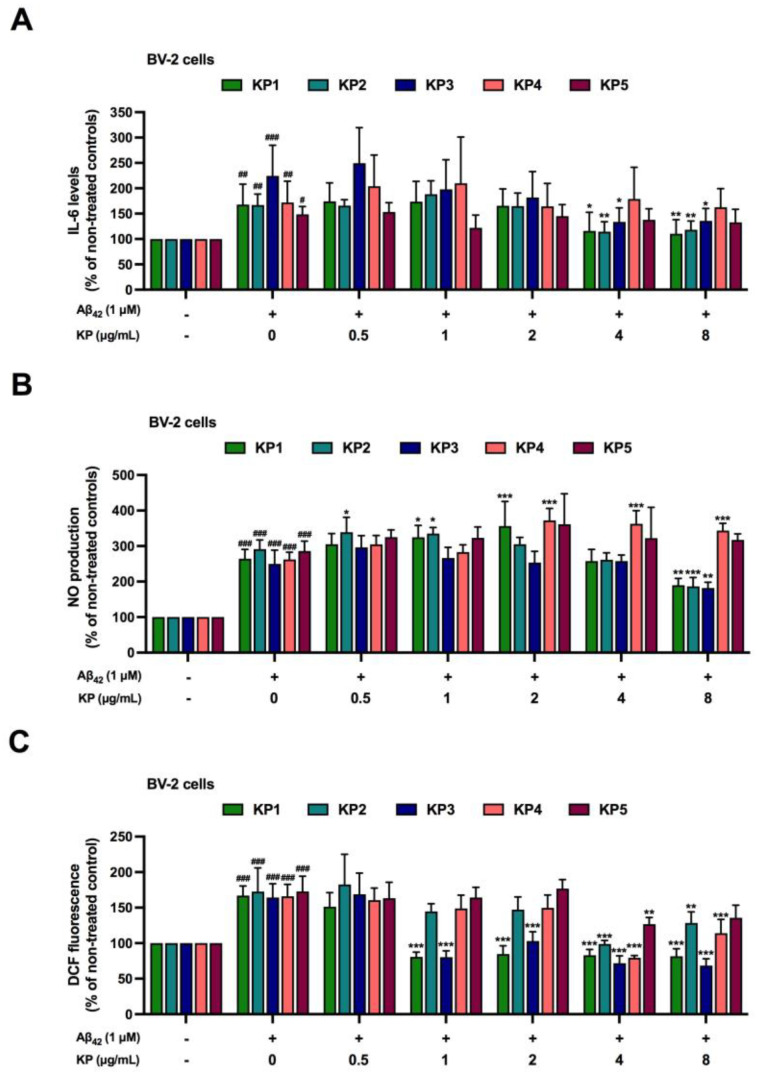
Anti-inflammatory and antioxidant effects of five *K. parviflora* fractions on Aβ_42_-induced neurotoxicity in monoculture of BV-2 cells. (**A**) IL-6 levels, (**B**) nitric oxide (NO) production, and (**C**) DCF fluorescence. Values are mean ± SD of three independent experiments. The ^#^ shows a significant difference between the control and cells exposed to Aβ_42_ peptides without KP extract using one-way ANOVA followed by Tukey’s test. ^#^, *p* < 0.05, ^##^, *p* < 0.01, ^###^, *p* < 0.001. The * shows a significant difference between cells exposed to Aβ_42_ peptides without KP extract compared to cells exposed to Aβ_42_ peptides and various concentrations of KP extract (0.5–8 µg/mL) using one-way ANOVA followed by Tukey’s test. *, *p* < 0.05, **, *p* < 0.01, ***, *p* < 0.001.

Aβ_42_ leads to neuronal death by two mechanisms as induction of inflammation and oxidative stress [38,39]. Thus, to explore how *K. parviflora* extracts prevented BV-2 death from Aβ_42_ (Figure 3A,B), we investigated the anti-inflammatory and antioxidant properties of KP1–KP5. BV-2 cell monoculture was co-exposed with Aβ_42_ (1 µM) with or without each fraction of *K. parviflora* extract (KP1-KP5). To avoid excessive inflammation and apoptotic interference, we used Aβ_42_ at 1 µM because this was a subtoxic dose (not more than IC_20_, Figure 1A,B). Biomarker levels of inflammatory responses such as interleukin-6 (IL-6) and nitrite, and cellular ROS were evaluated. Figure 4A–C confirm previous findings that Aβ_42_, even at a subtoxic dose (1 µM), operated as a neurotoxic agent by inducing inflammation (Figure 4A,B) and oxidative stress (Figure 4C) [38,39]. Treatment with *K. parviflora* extract (KP1-KP5) showed that only KP1, KP2, and KP3 fractions significantly reduced IL-6 levels (4 and 8 µg/mL), nitrite (8 µg/mL), and DCF fluorescence levels (KP1 and KP3 from 1–8 µg/mL and KP2 at 4 and 8 µg/mL) in a monoculture of Aβ_42_-treated BV-2 cells. Therefore, KP1, KP2, and KP3 prevented Aβ_42_-induced microglial death due to their anti-inflammatory and antioxidant properties.

### 3.5. Suppression of Aβ_42_-Induced Inflammation and Oxidative Stress by K. parviflora Extracts in Co-Culture between Differentiated NE-4C and BV-2 Cells

**Figure 5:**
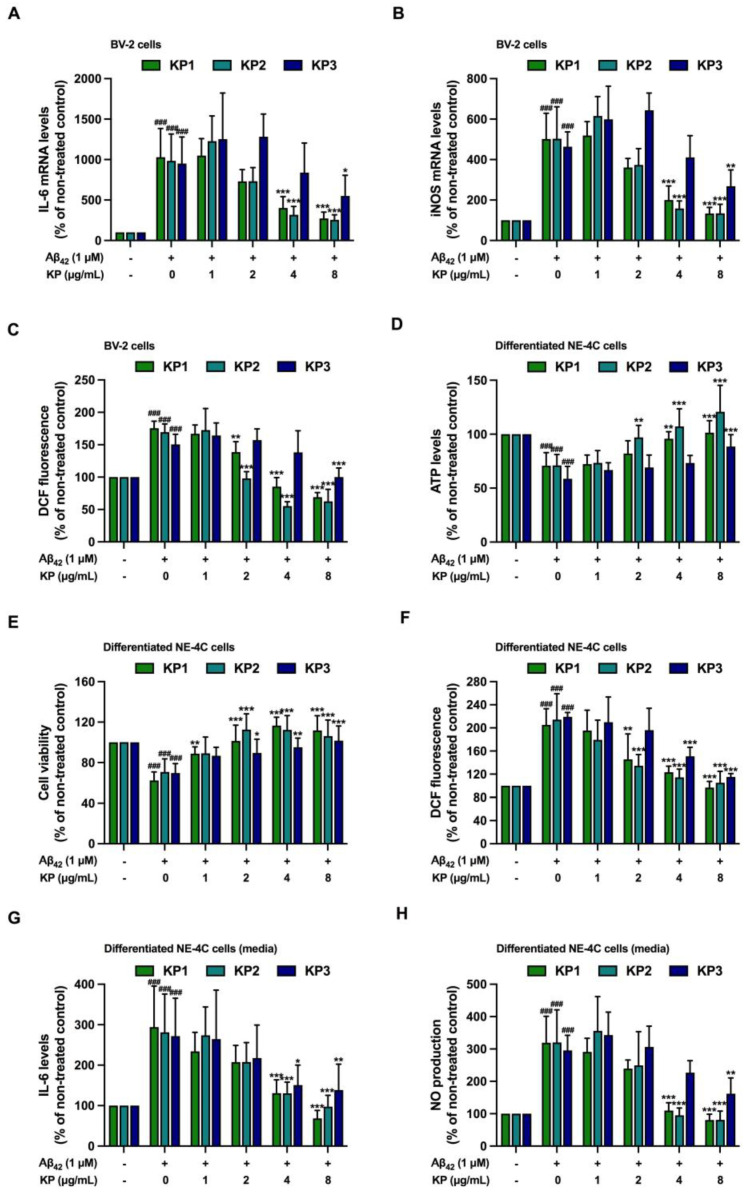
Anti-inflammatory and antioxidant effects of five *K. parviflora* fractions on Aβ_42_-induced neurotoxicity in co-culture between differentiated NE-4C and BV-2 cells. (**A**) IL-6 mRNA levels of BV-2 cells, (**B**) iNOS mRNA levels of BV-2 cells, (**C**) DCF fluorescence of BV-2 cells, (**D**) ATP levels of differentiated NE-4C, (**E**) cell viability of differentiated NE-4C, (**F**) DCF fluorescence of differentiated NE-4C, (**G**) IL-6 levels in culture media, and (**H**) NO production in the culture media. Values are mean ± SD of three independent experiments. The ^###^ shows significant difference between the control and cells exposed to Aβ_42_ peptides without KP extract at *p* < 0.001 using one-way ANOVA followed by Tukey’s test. The * shows significant difference between cells exposed to Aβ_42_ peptides without KP extract compared to cells exposed to Aβ_42_ peptides and various concentrations of KP extract (1–8 µg/mL) using one-way ANOVA followed by Tukey’s test. *, *p* < 0.05, **, *p* < 0.01, ***, *p* < 0.001.

Inflamed microglia eventually lead to neuronal dysfunction and death. Figure 3 and Figure 4 show the protective effects of KP1, KP2, and KP3 against neuronal death, inflammation, and oxidative stress in microglial BV-2 cell monoculture. Thus, the anti-inflammation and antioxidative stress of KP1, KP2, and KP3 fractions were further studied in co-culture between differentiated NE-4C and BV-2 cells. IL-6 and inducible nitric oxide synthase (iNOS) mRNA levels and cellular ROS levels in BV2-cells were significantly induced after Aβ_42_ treatment, confirming the neurotoxic effect of Aβ_42_ peptides (Figure 5A–C). However, these markers were reduced when KP1 and KP2 at 4–8 µg/mL and KP3 at 8 µg/mL were applied. 

The effects of Aβ_42_ peptides on differentiated NE-4C cells were also investigated. In the co-culture, Aβ_42_ peptides markedly decreased cell viability and caused oxidative stress in differentiated NE-4C cells compared to the untreated control (Figure 5D–F), suggesting the occurrence of inflamed microglia-mediated neuronal death. KP1 and KP2 (2, 4, and 8 µg/mL) and KP3 (4 and 8 µg/mL) prevented neuronal death and oxidative stress in differentiated NE-4C cells. Figure 5G,H show that IL-6 and nitrite levels in the cell culture medium were dramatically induced by Aβ_42_ peptides, whereas levels were significantly reduced by KP1, KP2, and KP3 treatments. Therefore, Aβ_42_ peptides caused inflammation and oxidative stress in co-culture between microglia and differentiated NE-4C cells leading to neuronal death. Interestingly, KP fractions KP1, KP2, and KP3 significantly decreased inflamed microglia and improved neuronal viability under Aβ_42_ induction. 

### 3.6. Suppression of Aβ_42_-Induced Inflammation and Oxidative Stress by K. parviflora Extracts in Co-Culture between Undifferentiated NE-4C and BV-2 Cells

Figure 5 shows the effects of inflamed microglia on differentiated NE-4C death; however, the effects of inflammatory microglia on neural stem cells that eventually develop into functional neurons remain unclear. Hence, the results of Aβ_42_-mediated inflamed microglia on undifferentiated NE-4C cells using the same co-culture strategy were further elucidated. 

**Figure 6:**
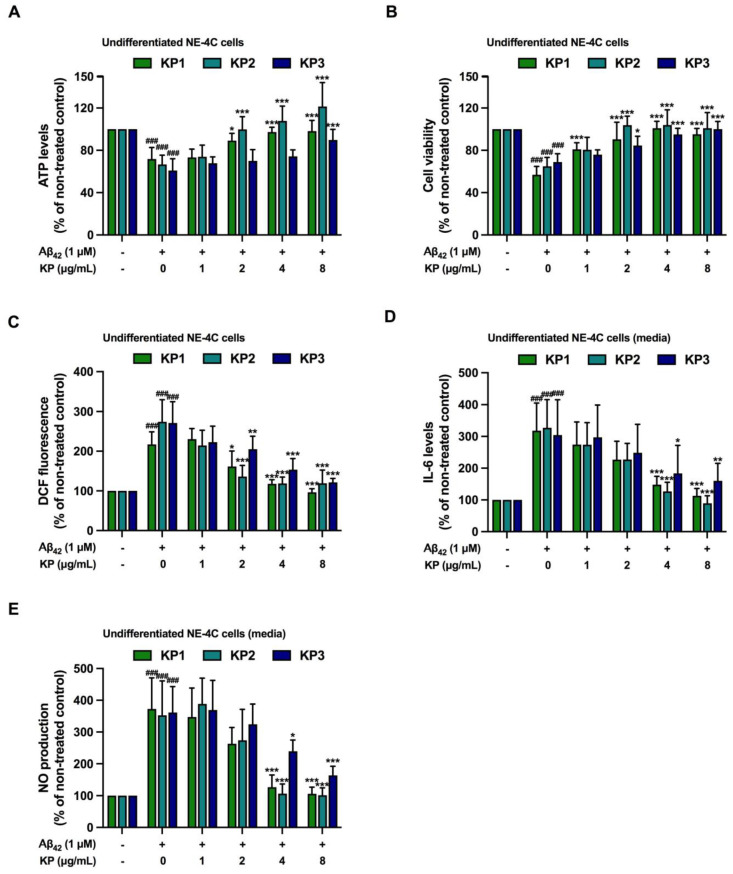
Anti-inflammatory and antioxidant effects of five *K. parviflora* fractions on Aβ_42_-induced neurotoxicity in co-culture between undifferentiated NE-4C and BV-2 cells. (**A**) ATP levels of undifferentiated NE-4C, (**B**) cell viability of undifferentiated NE-4C, (**C**) DCF fluorescence of undifferentiated NE-4C, (**D**) IL-6 levels in culture media, and (**E**) NO production in the culture media. Values are mean ± SD of three independent experiments. The ^###^ shows a significant difference between the control and cells exposed to Aβ_42_ peptides without KP extract at *p* < 0.001 using one-way ANOVA followed by Tukey’s test. The * shows a significant difference between cells exposed to Aβ_42_ peptides without KP extract compared to cells exposed to Aβ_42_ peptides and various concentrations of KP extract (1–8 µg/mL) using one-way ANOVA followed by Tukey’s test. *, *p* < 0.05, **, *p* < 0.01, ***, *p* < 0.001.

Figure 5A–C illustrate that Aβ_42_ peptides activated IL6 and iNOS mRNA expressions and oxidative stress (DCF fluorescence), while all inflammatory and oxidative stress markers were quenched by KP1, KP2, and KP3 extracts. Interestingly, inflamed BV-2 cells also decreased cell viability (Figure 6A,B), and caused oxidative stress (Figure 6C) and inflammation (Figure 6D,E) of undifferentiated neural stem cells in the same manner as differentiated neurons (Figure 5D–F). Treatment with KP extracts (KP1-KP3) protected against neuronal death and decreased oxidative stress (Figure 6A–E). These data implied that (i) Aβ_42_-mediated inflamed microglia led to the cytotoxicity of differentiated neurons and also undifferentiated neural stem cells, and (ii) KP1–KP3 decreased inflamed microglia, which subsequently resulted in improved viability of undifferentiated neural stem cells.

### 3.7. Protective Effects of K. parviflora Extracts on Neurogenesis of Aβ_42_-Treated NE-4 Cells in Monoculture

**Figure 7:**
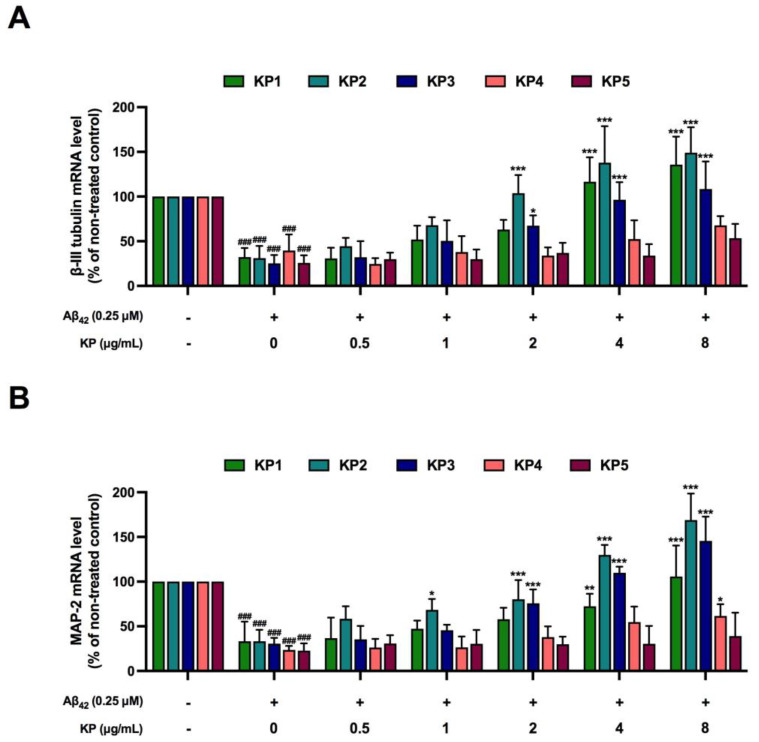
The protective effects of *K. parviflora* against neurogenesis-inhibiting Aβ_42_ peptides in monoculture of differentiated NE-4C cells. (**A**) beta-III tubulin mRNA levels and (**B**) MAP-2 mRNA levels. The ^###^ shows a significantly different between control and cells exposed to Aβ_42_ peptides without KP extract at *p* < 0.001 using one-way ANOVA followed by Tukey’s test. The * shows a significantly different between cells exposed to Aβ_42_ peptides without KP extract compared to cells exposed to Aβ_42_ peptides and various concentrations of KP extract (0.5–8 µg/mL) using one-way ANOVA followed by Tukey’s test. *, *p* < 0.05, **, *p* < 0.01, ***, *p* < 0.001.

Data in Figure 6 show that Aβ_42_ peptides reduced the viability of neural stem cells. Protective effects of KP extracts on neurogenesis-inhibiting Aβ_42_ peptide-treated cells were further elucidated by studying the expressions of two neuron-specific proteins, namely class III β tubulin (beta-III tubulin) and microtubule-associated protein 2 (MAP-2) in monoculture of differentiated NE-4 cells. Reductions in both beta-III tubulin and MAP2 mRNA levels were obtained when differentiated NE-4 cells were exposed to Aβ_42_ peptides (Figure 7A,B), suggesting that Aβ_42_ peptides directly inhibit neurogenesis, even at a nontoxic dose (0.25 µM, Figure 1A,B). Cells receiving both Aβ_42_ and various concentrations of KP fractions (KP2 and KP3 starting at 2 µg/mL, while KP1 starting at 4 µg/mL) showed significant recovery of beta-III tubulin and MAP-2 mRNA expressions, albeit at different potency. KP1, KP2, and KP3 fractions showed promising protective effects against neurogenesis-inhibiting Aβ_42_ peptides in the monoculture of differentiated NE-4 cells (Figure 7A,B).

### 3.8. Protective Effects of K. parviflora Extracts Neurogenesis in Aβ_42_-Induced Differentiated NE-4C in Co-Culture with BV-2 Cells

**Figure 8:**
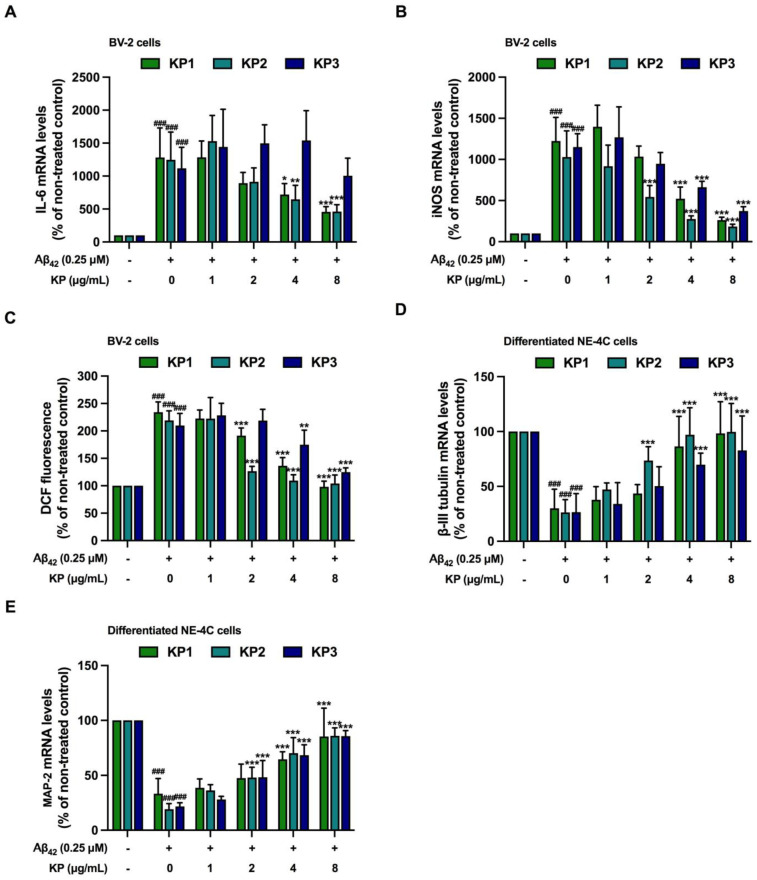
The protective effects of *K. parviflora* on neurogenesis in the co-culture between differentiated NE-4C and BV-2 cells treated with Aβ_42_. (**A**) IL-6 mRNA levels of BV-2 cells, (**B**) relative iNOS mRNA levels of BV-2 cells, (**C**) DCF fluorescence of BV-2 cells, (**D**) beta-III tubulin mRNA levels of differentiated NE-4C cells, and (**E**) MAP-2 mRNA levels of differentiated NE-4C cells. The values are mean ± SD of three independent experiments. The ^###^ shows a significantly different between control and cells exposed to Aβ_42_ peptides without KP extract at *p* < 0.001 using one-way ANOVA followed by Tukey’s test. The * shows a significantly different between cells exposed to Aβ_42_ peptides without KP extract compared to cells exposed to Aβ_42_ peptides and various concentrations of KP extract (1–8 µg/mL) using one-way ANOVA followed by Tukey’s test. *, *p* < 0.05, **, *p* < 0.01, ***, *p* < 0.001.

In addition to monoculture (Figure 7), we further investigated the protective effects of *K. parviflora* extracts against neurogenesis-inhibiting Aβ_42_ peptides in co-culture between differentiated NE-4C and BV-2 cells. Figure 8A–C show that without KP extracts, Aβ_42_ peptides induced inflammation and oxidative stress in BV-2 cells, similar to previous data (Figure 5A–C). Reduced inflammation and oxidative stress were also observed when each KP extract was added. Consistent with results obtained from undifferentiated cells shown in Figure 7, differentiated NE-4C cells exposed to Aβ_42_ peptides showed a reduction of both beta-III tubulin and MAP-2 mRNA expressions in co-culture (Figure 8D,E), suggesting that Aβ_42_ peptides not only inhibited neurogenesis directly but also reduced neurogenesis indirectly through inflamed microglia. 

Protective effects of the promising KP extracts KP1, KP2, and KP3 against neurogenesis-inhibiting Aβ_42_ peptides were also investigated. Intriguingly, protection indicated by the induction of beta-III tubulin and MAP-2 mRNA expressions was observed in all three KP fractions at 4 and 8 µg/mL (Figure 8D,E). These data indicated that KP1, KP2, and KP3 improved neurogenesis against Aβ_42_ peptide treatment.

## 4. Discussion

Alzheimer’s disease (AD) induces gradual cognitive impairment, with major pathological features such as the deposition of extracellular amyloid beta (Aβ) plaques and intercellular neurofibrillary tangles of tau protein. Microglial activation and neuroinflammation by Aβ plaques have been proposed as underlying and connecting components in the development of AD [2,3,4,40]. The involvement of stem cells in adult neurogenesis is critical for memory and cognitive performance [41]. In AD cases, Aβ plaque impairs the proliferation and differentiation of both neural stem cells and neurons [5,6,7] due to excessive induction of neuronal death and oxidative stress [6,7]. Therefore, limiting this vicious cycle by suppressing Aβ toxicity and promoting differentiation and/or proliferation of neural stem cells by safety agents shows promise as a potential therapeutic strategy to delay AD progression. *K. parviflora* has shown promising neuroprotective activity in several previous studies [42,43,44]. An ethanolic extract of *K. parviflora* protected glutamate-induced cell injury in mouse hippocampal neuronal cells [43], while *K. parviflora* protected oxidative stress-related brain damage and memory deficit induced by focal cerebral ischemia in rats [44]. The anti-AD effects targeting AChE and Aβ formation of *K. parviflora* have been previously studied [45,46] but the protective impacts of *K. parviflora* on Aβ-induced neurotoxicity, neuroinflammation, and neurogenesis have not yet been examined. In this study, NE-4C neural stem cells (undifferentiated), BV-2 microglia, and NE-4C-derived neurons (differentiated) were used in both monoculture and co-culture of NE-4C neural stem cells and BV-2 microglia, and co-cultures of NE-4C derived neurons and BV-2 microglia, to assess the impact of *K. parviflora* fractions on neuroprotective and anti-neuroinflammatory Aβ_42_ challenges, and on neurogenesis processes compromised by Aβ_42_. 

The *K. parviflora* fractions were characterized as crude ethanolic (KP1), n-hexane (KP2), chloroform (KP3), ethyl acetate (KP4) and residue (KP5). 5,7-Dimethoxyflavone (DMF) was detectable in KP3, KP1, KP2, and KP4, while 3,5,7,3’,4’-pentamethoxyflavone (PMF) and 5,7,4’-trimethoxyflavone (TMF) were found in KP1, KP3, and KP4. DMF was the primary bioactive phytochemical with antioxidant and anti-inflammatory activities [47,48,49,50,51]. DMF is used as a quality control marker, with KP powder produced for food ingredients containing at least 2.5% [52]. Our crude extract of KP contained 10.3% more DMF than the recommendation, while DMF, TMF, and PMF were absorbed through the GI tract and distributed throughout the brain, suggesting that these methoxyflavones, especially DMF, may offer neuroprotection [53,54] by inhibiting oxidative stress and neuroinflammation. 

Aβ_42_ generated cytotoxicity and elevated intracellular ROS in NE-4C neural stem cells and NE-4C derived neurons, while toxicity was greatly amplified when these cells were co-cultured with microglial cells. Co-exposure of Aβ_42_ at 5 μM with each KP fraction showed that crude ethanolic (KP1), n-hexane (KP2), and chloroform (KP3) fractions significantly decreased toxicity in a concentration-dependent manner in both mono- and co-cultures of BV-2, neural NE-4C stem cells, and neuronal-derived NE-4C cells (Figure 3). KP extract had neuroprotective impacts on mice hippocampus neuronal cells by promoting brain-derived neurotrophic factor (BDNF) expression and the extracellular signal-regulated kinase pathway (ERK) [43]. Furthermore, these three fractions demonstrated anti-inflammation triggered by Aβ_42_ in both BV-2 monoculture and co-culture (Figure 4, Figure 5 and Figure 6), with attenuated IL-6 and NO levels in the culture medium. These bioactive fractions reduced microglial activation by decreasing inducible nitric oxide synthase (iNOS) mRNA expression and intracellular ROS. NE-4C and neuron cells co-cultured with microglia were also protected by *K. parviflora* fractions from Aβ_42_, with boosted ATP levels and cell viability, while reducing intracellular ROS. *K. parviflora* has been shown to have anti-inflammatory properties that suppress the production of IL-6 in lipopolysaccharide-stimulated monocytes by inhibiting the activation of ERK1/2 and protein kinase B (Akt) [14,15]. Taken together, *K. parviflora* KP1, KP2, and KP3 fractions containing DMF protected toxicity in BV-2, neural NE-4C stem cells, and neuronal-derived NE-4C cells induced by Aβ_42_ peptides, while also protecting against cellular damage induced by microglial activation, as characteristics attributed to DMF [49,50,55]. 

To study the impact of KP extracts on Aβ_42_-disrupted neurogenesis, a low concentration of Aβ_42_ (0.25 µM) together with KP extract was applied in a monoculture of NE-4C cells for six days during 12 days of differentiation (Figure 7). The stated exposure period aimed was to assess how Aβ_42_ and the extracts impacted early differentiation before neuronal maturation [56]. The three KP fractions (KP1, KP2, and KP3) inhibited the action of Aβ_42_ and reversed the expression of specific neuronal mRNA markers, βIII-tubulin and MAP-2. The extracts reduced Aβ_42_-driven IL-6 and iNOS mRNA levels as well as intracellular ROS in microglial cells, coupled with lower IL-6 and NO levels in the coculture medium (Figure 8). Concerning differentiated neurons from NE-4C cells cocultured with microglia cells, the extracts also restored impaired neuronal development from Aβ_42_ in a similar way to the NE-4C cell monoculture. No data exist to demonstrate the influence of *K. parviflora* on neural stem cell differentiation but *K. parviflora* protected the rat brain against valproic acid-induced impairments in spatial memory and neural stem cell proliferation in the dentate gyrus [20]. These findings showed that *K. parviflora* extracts prevented the influence of Aβ_42_ on neurogenesis both directly on the functioning of NE-4C cells and indirectly by suppressing microglia-induced neuroinflammation. Apart from antioxidative and anti-inflammatory activities, neurogenesis signal transduction pathways involve ERK1/2, protein kinase A (PKA), Akt, WNT/β-catenin, protein kinase C (PKC), and BDNF [10,12]. Further research into molecular targets and the many biological neurogenetic activities of *K. parviflora* should concentrate on the ERK1/2, PKA, Akt, and BDNF signaling pathways [15,43,57].

The biological and toxic properties of methoxyflavones have generally focused on DMF. This study examined and compared the anti-inflammation and cytotoxicity of DMF, TMF and PMF in human dermal fibroblasts. TMF exhibited the highest cytotoxicity, while PMF did not influence on cell viability. TMF was the most effective polymethoxyflavone in decreasing TNF-α induced fibroblast damage through the MAPK and NF-κB pathways [58], while PMF was nontoxic and protective against oxidative DNA damage in RAW 264.7 macrophages [59]. Limited evidence exists on the anti-neuroinflammation, neuroprotection, and neurogenesis of DMF, TMF, and PMF individually. Current research is unable to distinguish their activity in each KP fraction or compare their efficacy. Further research is required to determine which methoxyflavone contributes to neuroinflammation and neurogenesis. Moreover, whether the neuroprotective mechanism of the KP extract and its active compound(s) is related to a decrease in ROS or other off-target effects should be investigated further.

## 5. Conclusions

Our results revealed that *K. parviflora* extracts protected against Aβ_42_ induced cellular damage of NE-4C neural stem cells, BV-2 microglia, and NE-4C derived neurons and also reduced microglia-induced cellular damage in NE-4C neural stem cells and neurons by Aβ_42_. Moreover, *K. parviflora* extracts also inhibited the inhibitory effects of Aβ_42_ on neuronal differentiation. These findings provide clues about the role of *K. parviflora* extracts in neuroprotection and neurogenesis and curing Aβ_42_-dependent AD. However, further detailed investigations are required to explore the molecular pathways governing neuroprotective actions against Aβ_42_ and the stimulatory effects in the neurogenesis of *K. parviflora* extracts.

## Figures and Tables

**Table 1:** Quantification of phytochemicals, DMF, PMF, and TMF contents in *K. parviflora* crude extract and its fractions.

Detected Compounds (mg/g Extract)	Crude Ethanolic Extract (KP1)	KP Fractions			
		Hexane (KP2)	Chloroform (KP3)	Ethyl Acetate (KP4)	Residue (KP5)
Caffeic acid	ND	ND	ND	4.07 ± 0.91	ND
Catechin	ND	ND	ND	ND	15.6 ± 1.40
Rutin	ND	ND	ND	13.4 ± 2.80	ND
DMF	103.27 ± 1.15	8.75 ± 0.17	132.40 ± 0.01	2.90 ± 0.05	ND
PMF	123.95 ± 38.83	2.74 ± 0.34	156.64 ± 0.14	23.34 ± 0.02	ND
TMF	517.19 ± 2.10	35.19 ± 3.31	649.54 ± 6.67	76.09 ± 0.26	ND

The value is represented as mean ± SD. ND.: non-detectable. DMF: 5,7-dimethoxyflavone, PMF: 3,5,7,3′,4′-pentamethoxyflavone, and TMF: 5,7,4′-trimethoxyflavone.

## Data Availability

The original contributions presented in the study are included in the article/Supplementary Material.

## Supplementary Materials

The following supporting information can be downloaded at: https://www.mdpi.com/article/10.3390/nu15051098/s1. Supplementary Table S1. Primers used for real-time PCR; Supplementary Figure S1. HPLC chromatogram for determination of catechin, caffeic acid, rutin, rosmarinic acid, quercetin, apigenin and kaempferol in (A) crude ethanolic extract (KP1), and (B) hexane (KP2), (C) chloroform (KP3), (D) ethyl acetate (KP4), and (E) residue (KP5) fractions, (F) shows the HPLC fingerprint of a standard mixture including, (A) catechin, (B) caffeic acid, (C) rutin, (D) rosmarinic acid, (E) quercetin, (F) apigenin, (G) kaempferol, and (H) 5,7-dimethylflavone; Supplementary Figure S2. HPLC chromatogram for determination of flavone derivatives including PMF (3,5,7,3’,4’-pentamethoxyflavone), DMF (5,7-dimethoxyflavone) and TMF (5,7,4’-trimethoxyflavone) contents in (A) crude ethanolic extract (KP1), and (B) hexane (KP2), (C) chloroform (KP3), (D) ethyl acetate (KP4), and (E) residue (KP5) fractions, (F) shows the HPLC fingerprint of a standard mixture of PMF, DMF, and TMF.
