# Supplementary material for: Kaempferia parviflora Extracts Protect Neural Stem Cells from Amyloid Peptide-Mediated Inflammation in Co-Culture Model with Microglia
Source: Nutrients. 2023 Feb 22;15(5):1098. doi: 10.3390/nu15051098 (PMC10004790; doi:10.3390/nu15051098)

nutrients

MDPI

Supplementary materials

Kaempferia parviflora Extracts Protect Neural Stem Cells from Amyloid Peptide-Mediated Inflammation in Co-Culture Model with Microglia

Piya Temviriyanukul [1], Anchana Chansawhang [2], Jirarat Karinchai [3], Sataporn Phochantachinda [4], Shutipen Buranasinsup [5], Woorawee Inthachat [1], Pornsiri Pitchakarn [3,*] and Boonrat Chantong [5,*]

[1] Food and Nutrition Academic and Research Cluster, Institute of Nutrition, Mahidol University, Salaya, Phuttamonthon, Nakhon Pathom 73170, Thailand; piya.tem@mahidol.ac.th (P.T.); woorawee.int@mahidol.ac.th (W.I.)
[2] The Center for Veterinary Diagnosis, Faculty of Veterinary Science, Mahidol University, Salaya, Phutthamonthon, Nakhon Pathom 73170, Thailand; anchana.chn@mahidol.ac.th (A.C.)
[3] Department of Biochemistry, Faculty of Medicine, Chiang Mai University, Chiang Mai 50200, Thailand; jirarat.ka@cmu.ac.th
[4] Department of Clinical Sciences and Public Health, Faculty of Veterinary Science, Mahidol University, Salaya, Phutthamonthon, Nakhon Pathom 73170, Thailand; sataporn.pho@mahidol.ac.th (S.B.)
[5] Department of Pre-clinic and Applied Animal Science, Faculty of Veterinary Science, Mahidol University, Salaya, Phuttamonthon, Nakhon Pathom 73170, Thailand; shutipen.bur@mahidol.ac.th

* Correspondence: pornsiri.p@cmu.ac.th (P.P.); boonrat.cha@mahidol.ac.th (B.C.)

Supplementary Table S1:

Primers used for real-time PCR.

Genes	Primers	Sequences
GAPDH	forward	CTCGTGGAGTCTACTGGTGT
(mouse)	reverse	GTCATCATACTTGGCAGGTT
iNOS	forward	ATGAGGTACTCAGCGTGCTCCAC
(mouse)	reverse	CCACAATAGTACAATACTACTTGG
IL-6	forward	GGAGGCTTAATTACACATGTT
(mouse)	reverse	TGATTTCAAGATGAATTGGAT
βIII-tubulin	forward	ACCCCGTGGGCTCAAAAT
(mouse)	reverse	CCGGAACATGGCTGTGAACT
MAP-2	forward	CCTGGTGCCCAGTGAGAAGA
(mouse)	reverse	GTCCGGCAGTGGTTGGTTAA

Supplementary Figure S1:

HPLC chromatogram for determination of catechin, caffeic acid, rutin, rosmarinic acid, quercetin, apigenin and kaempferol in (A) crude ethanolic extract (KP1), and (B) hexane (KP2), (C) chloroform (KP3), (D) ethyl acetate (KP4) and (E) residue (KP5) fractions. Supplementary Figure S1 (F) shows the HPLC fingerprint of standard mixture including, (A) catechin, (B) caffeic acid, (C) rutin, (D) rosmarinic acid, (E) quercetin, (F) apigenin, (G) kaempferol, and (H) 5,7-dimethylflavone.

(A) Crude ethanolic extract (KP1)

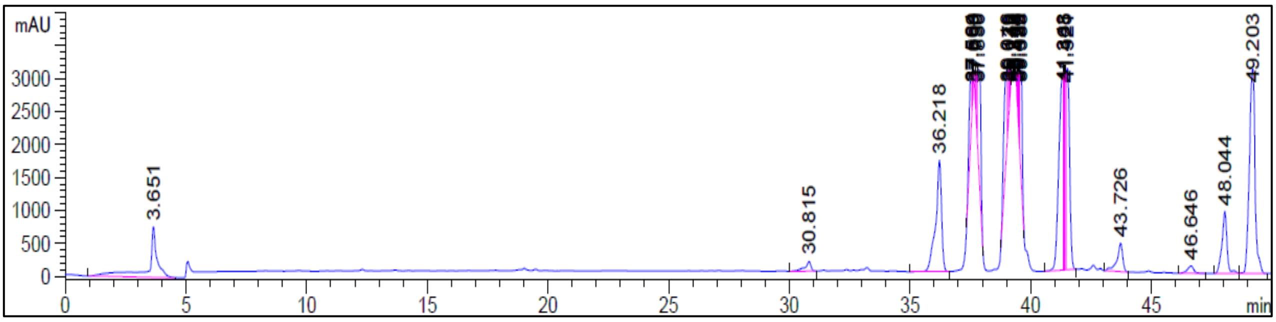

(B) Hexane fraction (KP2)

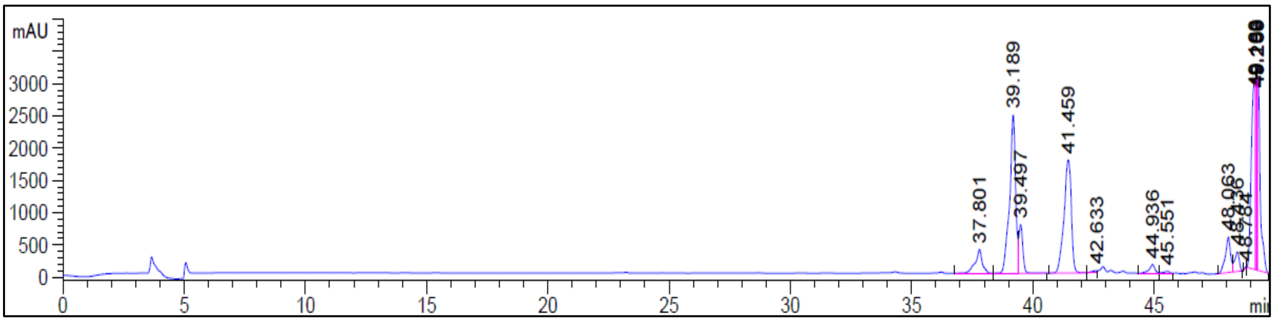

(C) Chloroform fraction (KP3)

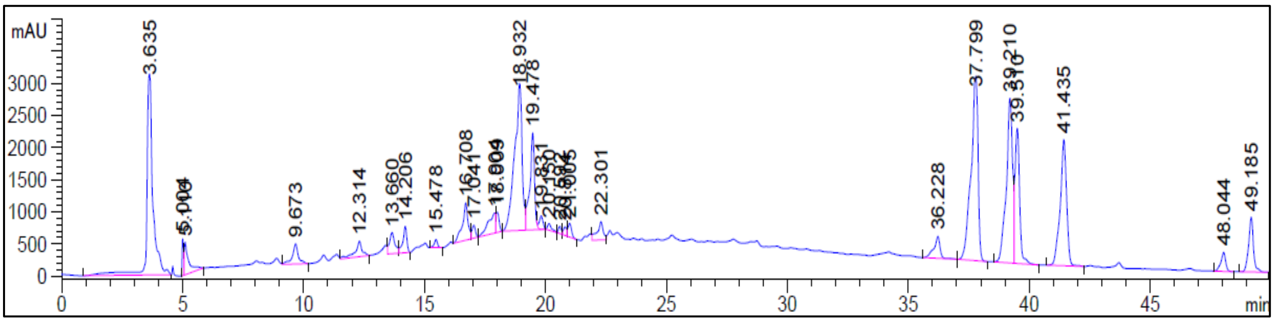

Supplementary Figure S1 (Cont.):

HPLC chromatogram for determination of catechin, caffeic acid, rutin, rosmarinic acid, quercetin, apigenin and kaempferol in (A) crude ethanolic extract (KP1), and (B) hexane (KP2), (C) chloroform (KP3), (D) ethyl acetate (KP4) and (E) residue (KP5) fractions. Supplementary Figure S1 (F) shows the HPLC fingerprint of standard mixture including, (A) catechin, (B) caffeic acid, (C) rutin, (D) rosmarinic acid, (E) quercetin, (F) apigenin, (G) kaempferol, and (H) 5,7-dimethylflavone.

(D) Ethyl acetate fraction (KP4)

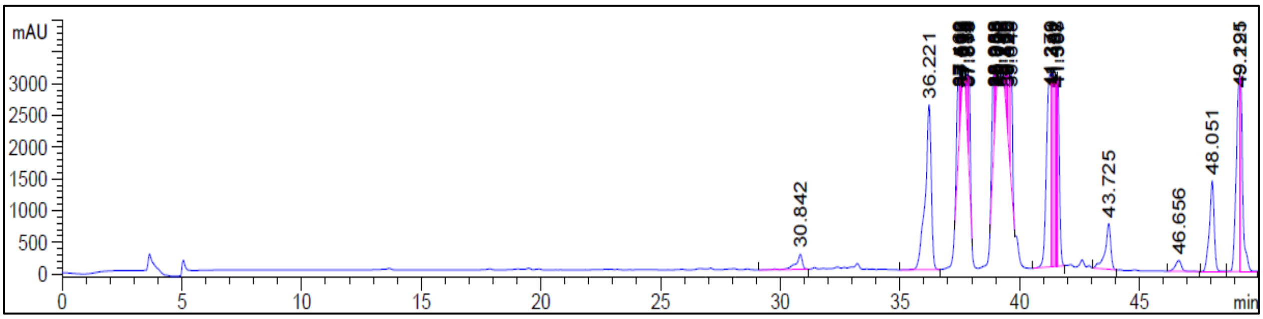

(E) Residue (KP5)

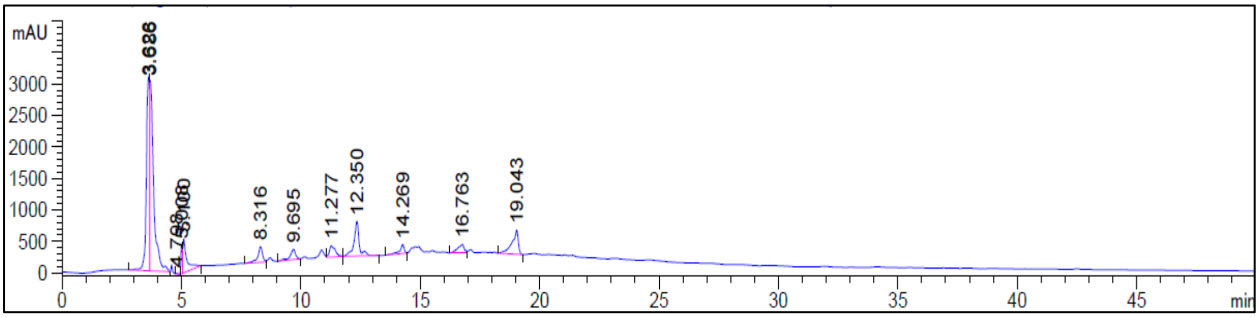

(F) Standard mixture

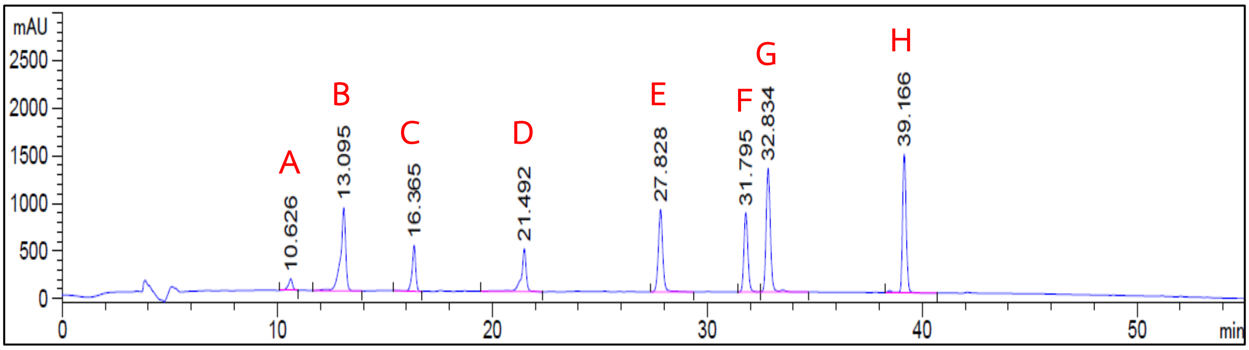

Supplementary Figure S2:

HPLC chromatogram for determination of flavone derivatives including PMF (3,5,7,3',4'-pentamethoxyflavone), DMF (5,7-dimethoxyflavone) and TMF (5,7,4'-trimethoxyflavone) contents in (A) crude ethanolic extract (KP1), and (B) hexane (KP2), (C) chloroform (KP3), (D) ethyl acetate (KP4) and (E) residue (KP5) fractions. Supplementary Figure S2 (F) shows the HPLC fingerprint of standard mixture of PMF, DMF, and TMF.

(A) Crude ethanolic extract (KP1)

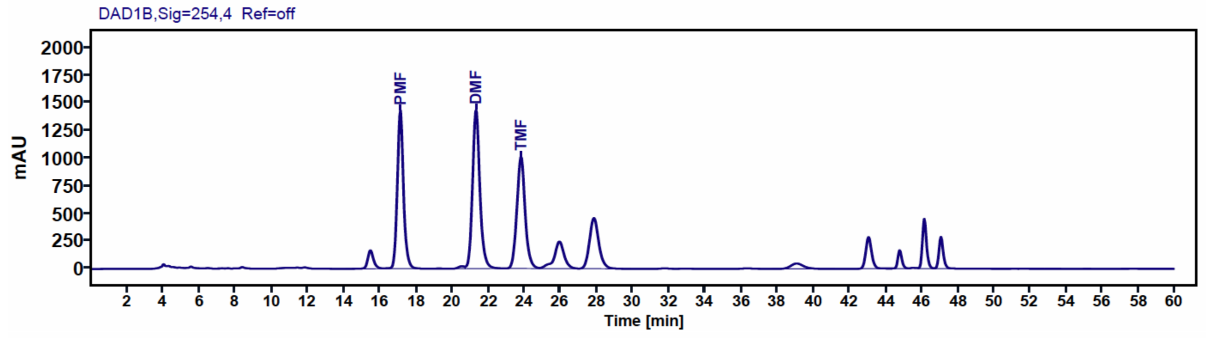

(B) Hexane fraction (KP2)

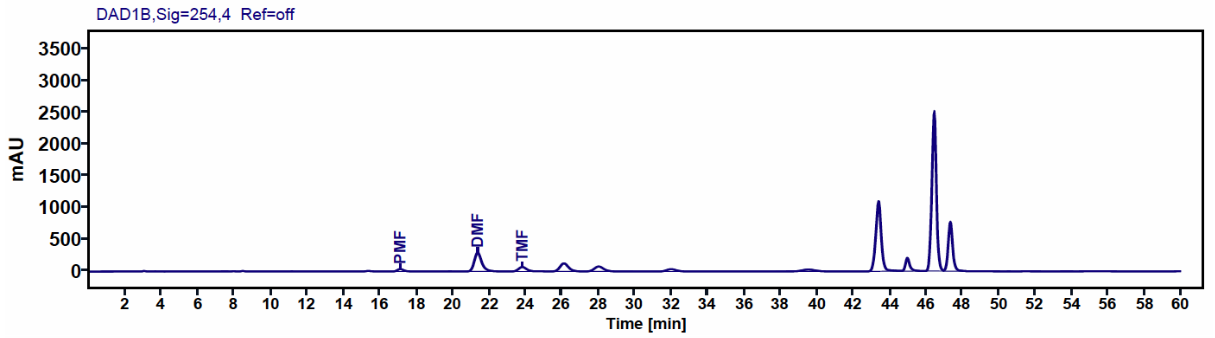

©

(C) Chloroform fraction (KP3)

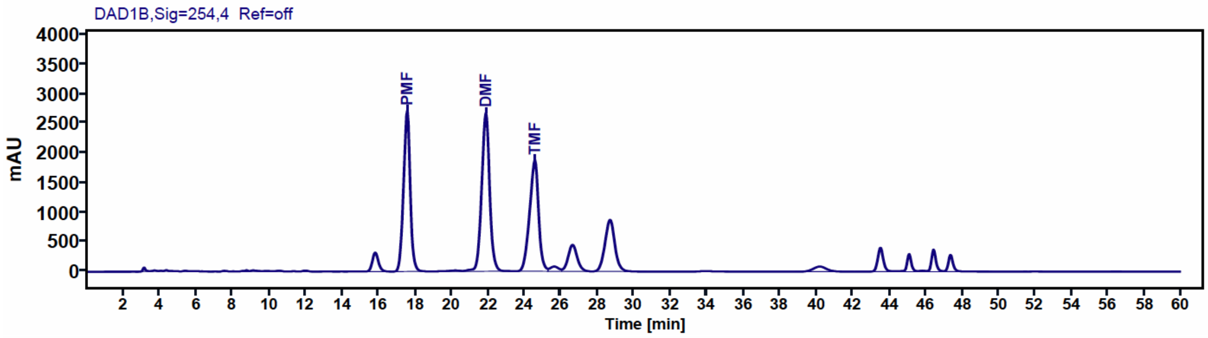

Supplementary Figure S2 (Cont.):

HPLC chromatogram for determination of flavone derivatives including PMF (3,5,7,3',4'-pentamethoxyflavone), DMF (5,7-dimethoxyflavone) and TMF (5,7,4'-trimethoxyflavone) contents in (A) crude ethanolic extract (KP1), and (B) hexane (KP2), (C) chloroform (KP3), (D) ethyl acetate (KP4) and (E) residue (KP5) fractions. Supplementary Figure S2 (F) shows the HPLC fingerprint of standard mixture of PMF, DMF, and TMF.

(D) Ethyl acetate fraction (KP4)

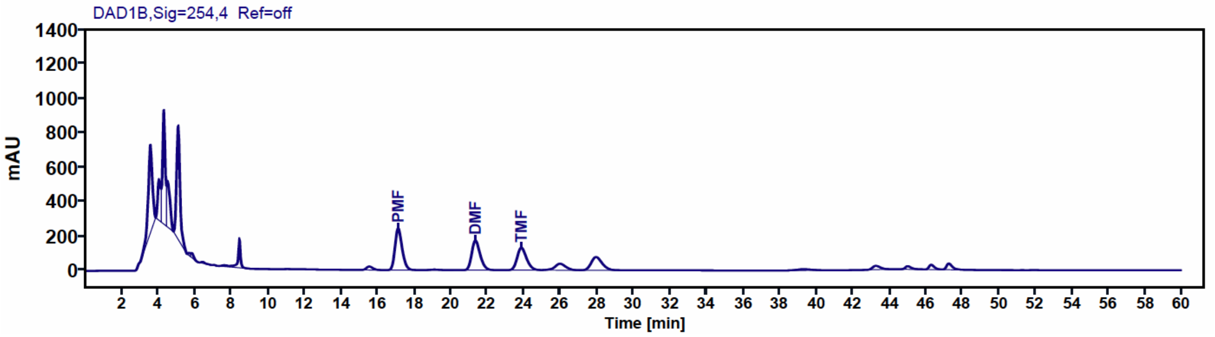

(E) Residue (KP5)

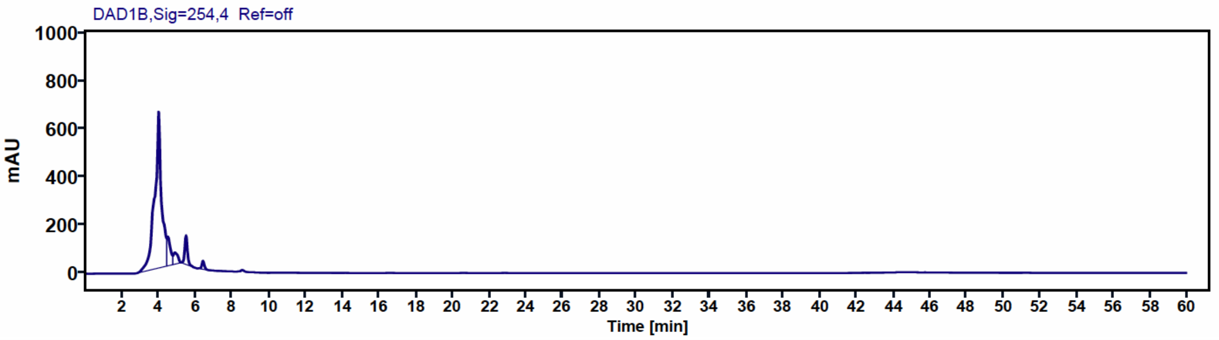

(F) Standard mixture

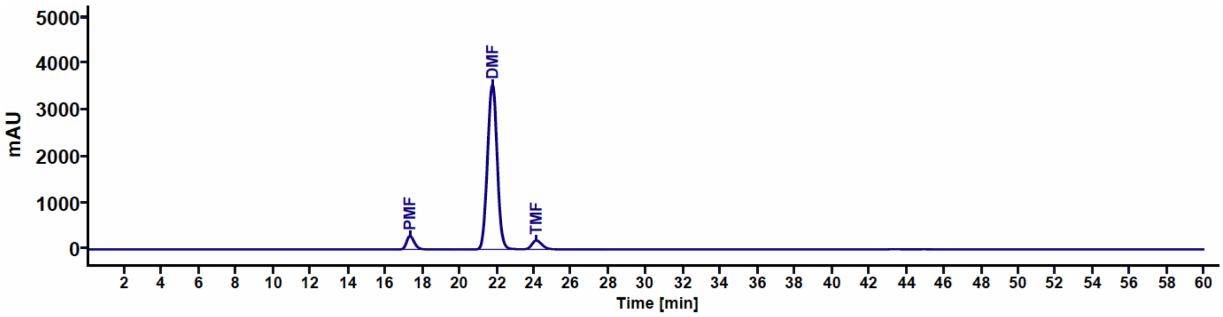

Supplement: Supplementary file 1 [file nutrients-15-01098-s001.zip › nutrients-2217888-supplementary.pdf]
